# Isokinetic muscle strength cannot be related to the odds ratio of musculoskeletal injuries in young elite wrestlers

**DOI:** 10.1186/s13102-022-00423-3

**Published:** 2022-02-22

**Authors:** Alireza Hoseini, Mostafa Zarei, Hadi Nobari, Fariborz Hovanloo, Hamed Abbasi, Jorge Pérez-Gómez

**Affiliations:** 1 Department of Sport Rehabilitation and Health, Faculty of Sport Science and Health, Shahid Beheshti University, Tehran, Iran; 2 Department of Exercise Physiology, Faculty of Educational Sciences and Psychology, University of Mohaghegh Ardabili, Ardabil, 56199-11367 Iran; 3 Sepahan Football Club, Isfahan, 81887-78473 Iran; 4 Department of Sport Injuries and Corrective Exercises, Sport Sciences Research Institute, Tehran, Iran; 5 HEME Research Group, Faculty of Sport Sciences, University of Extremadura, 10003 Cáceres, Spain

**Keywords:** Isokinetic strength, Prediction of injuries, Wrestler, Elite, Muscles' strength ratio

## Abstract

**Background:**

Wrestling is considered one of the oldest sports in the world. There is a high rate of injuries in Wrestling. To prevent injuries, it is necessary to identify the risk factors. Despite the functional importance of strength in wrestling, few studies have investigated the relationship between isokinetic strength and the rate of injuries in the sport.

**Objectives:**

The objective of the present study was to investigate the relationship between the isokinetic strength of elite wrestlers and the rate of injuries prospectively.

**Method:**

A total of 72 young wrestlers with at least 2 years of experience in the Tehran Wrestling Premier League participated in this study. Before the start of the competition season, the isokinetic strength of flexor and extensor muscles of the knee and shoulder were measured at different velocities by an isokinetic dynamometer. The injuries and training hours of these wrestlers were then recorded for nine months.

**Results:**

The study results showed no significant relationship between isokinetic strength of flexor and extensor muscles of the knee or shoulder at different angular speeds of 60, 180, and 300°/s. There was neither significant between the ratio of the strength flexor/extensor for knee and/or shoulder in young elite wrestlers with injuries.

**Conclusion:**

Isokinetic strength of lower and upper limb muscles alone cannot predict wrestlers' injuries. Therefore, the use of these tests is not recommended to evaluate the risk of injury in this population.

## Background

Wrestling is one of the most popular sports in Iran and has its roots in Iranian history and traditions, so it is commonly referred to as the national sport of Iran. This sport has different styles, the most popular are Freestyle and Greco-Roman [1]. Formal and Olympic competitions are also held in these two styles. In Greco-Roman style wrestling, it is forbidden to take parts lower than the waist and use feet in performing techniques. In freestyle wrestling, it is possible to use one's foot and the opponent's foot in offensive and defensive situations [2].

Although wrestling increases physical fitness and self-confidence; the rate of injuries in this sport is also high [3]. Hootman et al. stated that the rate of wrestling and soccer injuries is higher than other sports among American students [4, 5]. Pasque et al. [6] also reported the rate of 9 injuries per 1000 h in high school wrestlers. Halloran et al. [7] estimated the rate of injuries among American high school and college freestyle wrestlers as 9.6 per 1000 h. The costs of injuries in wrestling are estimated at more than $ 650 million annually in the United States [5]. Therefore, to maintain the safety and health of wrestlers and to prevent waste of financial resources, it is necessary to prevent injuries in this sport.

Identifying risk factors is essential to prevent injuries. The researchers have identified a variety of risk factors for injuries in wrestling. For example, weight loss and dehydration [8], equipment used such as mattresses, earmuffs, mouth protectors [9], and insufficient monitoring of the instructor on training and experience of wrestlers have been mentioned as risk factors of wrestling [10]. For example, it has been stated that wrestlers had higher injury rates during weight loss periods than during other periods of training. Wrestling has different rates of injury and severities depending on weight class and wrestling style [11]. Also it has been reported that compared to female freestyle wrestlers, male freestyle wrestlers had higher injury rates. wrestling and weight class influenced injury severity [12].

Many researchers consider muscle strength and endurance as one of the most important factors for the success of elite wrestlers [13]. The wrestlers need maximum dynamic strength to perform defensive and offensive techniques. The techniques that lead to lifting and throwing the opponent require high strength. Isometric strength is also essential for wrestlers and opponent control [14]. Tatlici et al. stated that wrestling-specific training reduces the hamstring to quadriceps (H/Q) muscle strength rate and can rise the probability of knee injuries [15]. Uğur et al. [16] declared that as the strength of leg muscle increases, the injury risk decreases, it has also been reported that the proportion between hamstring and quadriceps muscles in freestyle wrestlers’ upper leg strength values is not ideal. Addressing that injury risk increases with loss of strength [17]. Studies have shown that eccentric quadriceps strength and low eccentric flexor/extensor ratio are related to an increase in anterior cruciate ligament injury risk, and emphecized that H/Q values should be determined to prevent injuries, and also for rehabilitation [18]. Despite the importance of strength in performance and injury prevention, limited studies have investigated the relationship between strength and injury in wrestlers. Therefore, the objective of the present study was to investigate the relationship between the isokinetic strength of elite male wrestlers and their rate of injuries prospectively.

## Material and method

### Participants

The statistical population of the present study was all-male wrestlers participating in the Tehran Wrestling Premier League. All clubs were invited to participate in this study. A total of 72 wrestlers (51 Freestyle wrestlers and 21 Greco-Roman wrestlers) participated in the study voluntarily. The sample size was estimated by G Power software with 95% power and OR equal to 2.5 based on a study by Bakken et al. [19]. Having at least 5 years of wrestling experience and at least 2 years of wrestling experience in the provincial premier league, age range under 21 years, no serious injury that has led to an absence of more than 28 days in the last six months, having the direction of a normal limb in the lower limbs and having at least three training sessions per week were the study inclusion criteria. Failure to participate in regular training, absence from 5 consecutive training sessions, and participation in regular injury prevention programs for 6 weeks were the study exclusion criteria. The ethics committee at Shahid Beheshti University in Tehran, Iran, gave their clearance before the start experimental, also in this time, all parents or guardian and subjects signature the consent form. All declarations of Helsinki were followed in this investigation.

### The experimental approach to the problem

This is a comparative and prospective study. The independent variables of this study included isokinetic strength of flexor and extensor muscles of the knee and shoulder. The dependent variable is the presence or absence of injury. The laboratory steps were performed after the wrestlers entered the laboratory, their height was first measured by Stadiometer inbody co: BSM370, and the weight and fat percent of the wrestlers were measured by Inbody 770 Model: bpm040s12fxx. To warm up the shoulder and knee muscles, the wrestlers trained on the ergometer bike for 10 min at a desired intensity and speed. The isokinetic strength of the superior shoulder and knee muscles were measured using Biodex isokinetic system pro 4 systems (CMVAG Con-Trex Co., Swiss). The isokinetic strength was measured in the morning from 9 to 12 o'clock.

### Data measurement and variables

#### Measurement of isokinetic strength of flexor and extensor muscles of the knee

**Fig. 1:**
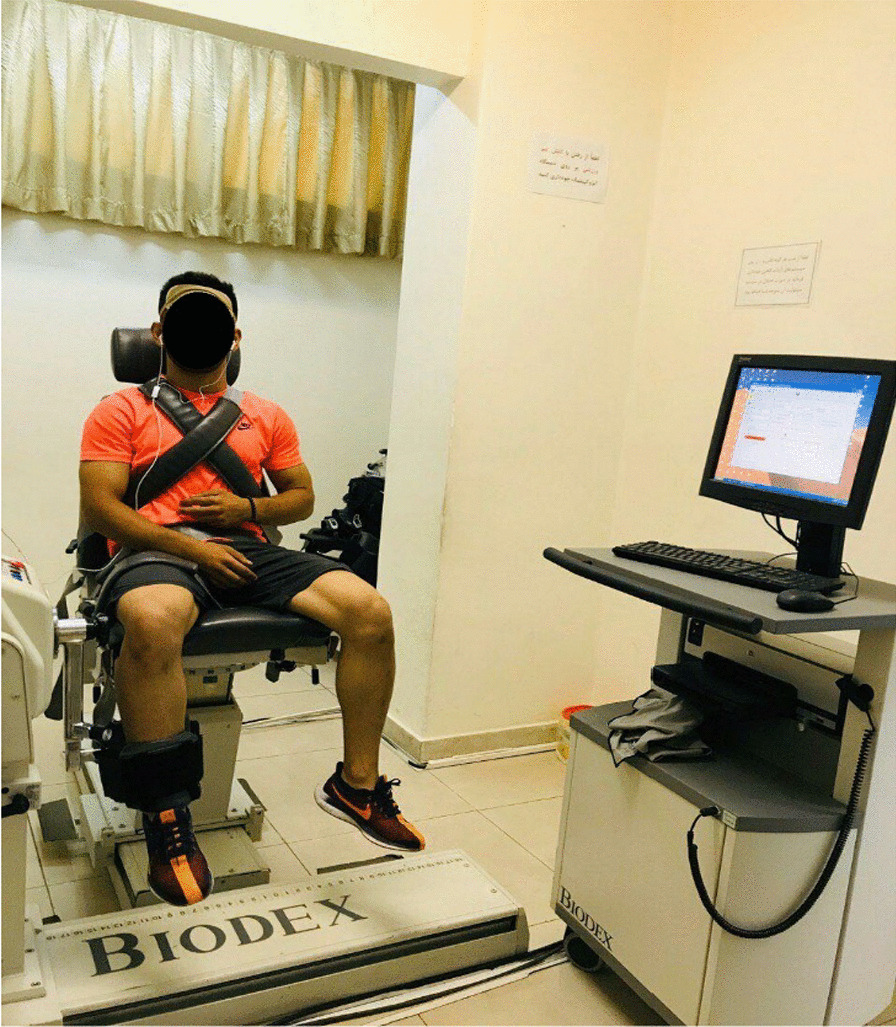
Measurement of isokinetic strength of the knee

All tests were performed by one of the researchers. All tests were conducted between 9 and 12 a.m. To measure concentric isokinetic to concentric strength of hamstring and quadriceps muscles, and eccentric isokinetic to eccentric strength of hamstring and quadriceps muscles in both positions, the subject was asked to sit on the device seat so that his body was in a comfortable and standard position. To perform the test in an optimal and standard way, the trunk, pelvis, and thigh of the foot were fixed using special belts on the device. According to the standards mentioned in the user manual of the isokinetic device, the rotations, height, and angles related to the position of the seat and the dynamometer were adjusted. The final settings were made as to the center of the rotation axis of the dynamometer and the center of the axis of rotation of the knee joint are matched. Then, the corresponding arm, which is specially designed for the left or right foot, was mounted on a dynamometer based on the test foot. After adjusting the height of the arm to the length of the foot, using a special cushion belt, the foot on the arm was closed and tightened so that the cushion was placed on the ankle. Wrestlers performed the tests with their dominant leg (i.e. the leg that athletes preferably kick the ball with). The subject was asked to perform a few natural contractions along with the range of motion, to ensure the comfort of the person, the correct performance of the movement as well as creating familiarity and communication between the person and the device, and then the subject was asked to try with maximum strength and speed at angular speeds of 60° (low intensity), 180° (moderate intensity) and 300° (high intensity) [20], respectively to perform flexion and extension movements of the knee in the range of 0°–90° according to the command of the device or tester [21]. We were looking to use to similar to the wrestling's movement patterns and the wrestling movements are based on low, medium, and high speeds. Therefore, we selected these velocities to measure isokinetic strength. In each of the angular speeds, the subject had the opportunity to try 5 times, after which he rested for 30 s, and rested for 1 min between movements at different angular speeds. The eccentric to eccentric strength was measured in the same way but at two angular speeds of 60° and 180° [22, 23] (Fig. 1).

#### Measurement of isokinetic strength of flexor and extensor muscles of the shoulder

Biodex System 4 isokinetic dynamometer was used to measure the isokinetic strength of the subjects' dominant shoulder flexor and extensor muscles. The subjects were placed on a special chair according to the standard provided by the device guide, his trunk was fixed by special belts, his superior shoulder was measured while sitting, and a special movement was attached to their upper limbs. To measure the maximum torque of flexor and extensor muscles, the subject moves his arm in the sagittal plane and performs 5 repetitions with the maximum effort in the range of 0–180 at three speeds of 60, 180, and 300°/s. In each of the angular speeds, the subject had the opportunity to try 5 times, after which he rested for 60 s, and another 3 min of recovery were performed between movements at different angular speeds. The best value was used for the analysis.

**Fig. 2:**
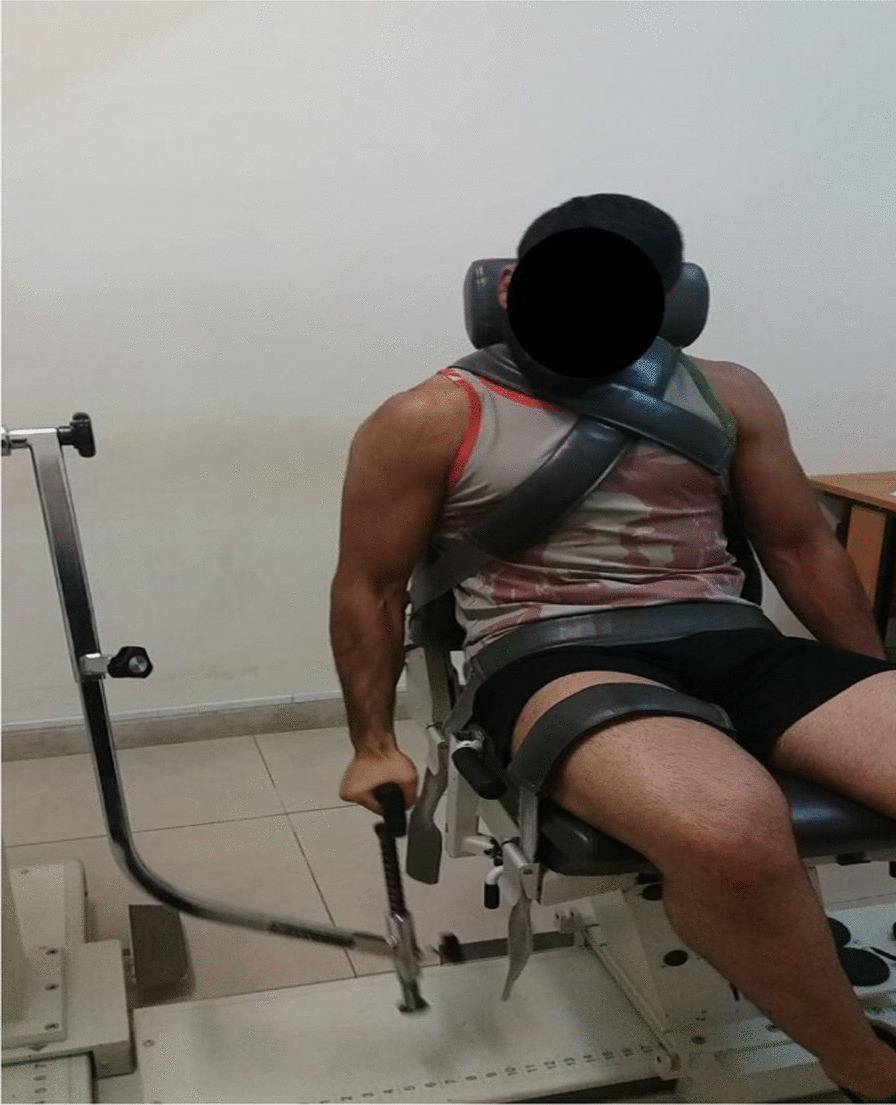
Measurement of shoulder isokinetic strength

The rest time was 1 min between each effort and 3 min between each exercise (Fig. 2). We used the Edinburgh Handedness Inventory questionnaire to determine the hand preference of the wrestler [24].

#### Record of injuries and training hours

In this study, an injury was recorded when (1) occurred while participating in training or competitions, (2) need medical help (medical team to reach the wrestler), and (3) the injured wrestler is not able to participate in training session or competition the next day [25]. Wrestling training hours and competitions were also recorded in a special form.

### Statistical analysis methods

The data obtained from the measurement of research variables were analyzed using SPSS software version 26 and descriptive and inferential statistics. Muscle strength measures were presented as absolute (for agonist/antagonist ratio tests) and body mass-normalized values for the other variables. We used the measurements as Peak Torque/Body Weight. The significance level was considered equal to 0.5 and alpha was less than or equal to 0.95. A logistic regression test was used to investigate the relationship between isokinetic strength and the rate of injuries.

## Results

Data from the 72 wrestlers were analyzed. Table 1 shows demographic and anthropometric data such as age, height, weight, body mass index, and body fat percent of wrestlers at the beginning of the study.

**Table 1:** Characteristics of all wrestlers (N = 72)

Variables	Value
Age (years)	19.3 ± 3.8
Height (cm)	174.3 ± 6.8
Body mass (kg)	80.2 ± 22.0
Body mass index (kg/m^2^)	25.3 ± 5.9
Percent body fat (%)	13.3 ± 5.8

Data are presented as mean ± standard deviation

A total of 72 wrestlers participated in this cohort study to investigate the relationship between some internal risk factors and musculoskeletal injuries of young wrestlers. During the 8 months of the study, 40 injuries were recorded. These injuries affected 34 wrestlers out of a total of 72 wrestlers (47%). 40% of injuries occurred in the lower limbs. 70% of injuries were acute and 30% were chronic.

The results of the logistic regression test showed that no significant relationship was found between the isokinetic strength of knee flexor muscles at different speeds of concentric and eccentric contractions and the rate of injuries in young wrestlers. However, wrestlers who had higher average strength to perform concentric flexion at different speeds of 60, 180, and 300°/s, and eccentric flexion at different speeds of 60 and 180°/s suffered significantly fewer injuries than other wrestlers (Table 2).

**Table 2:** Comparison logistic regression analysis between injured and non-injured wrestler (knee)

Variables	Non-Injured	Injured	Lower	Upper	OR	*P* value
Maximum isokinetic concentric flexion 60°/_S_	1.99 ± 0.63	1.69 ± 0.52	0.01	1.27	0.33	0.46
Maximum isokinetic concentric flexion 180°/_S_	1.48 ± 0.44	1.29 ± 0.40	0.08	1.04	0.37	0.08
Maximum isokinetic concentric flexion 300°/_S_	1.49 ± 0.52	1.25 ± 0.44	0.05	1.10	0.58	0.65
Maximum isokinetic eccentric flexion 60°/_S_	4.74 ± 1.21	4.26 ± 1.26	0.27	1.14	0.77	0.61
Maximum isokinetic eccentric flexion 180°/_S_	4.52 ± 1.42	4.07 ± 1.32	0.46	1.68	1.31	0.60
Maximum isokinetic concentric extension 60°/_S_	4.10 ± 1.11	3.61 ± 1.05	0.17	1.44	1.15	0.88
Maximum isokinetic concentric extension 180°/_S_	2.85 ± 0.72	2.52 ± 0.75	0.06	1.92	1.23	0.88
Maximum isokinetic concentric extension 300°/_S_	2.57 ± 0.73	2.19 ± 0.70	0.84	1.75	0.48	0.41
Maximum isokinetic eccentric extension 60°/_S_	3.00 ± 0.89	2.65 ± 0.86	0.26	2.34	1.64	0.59
Maximum isokinetic eccentric extension 180°/_S_	2.86 ± 0.88	2.53 ± 1.00	0.28	1.83	1.04	0.94
Maximum isokinetic concentric extension/flexion 60°/_S_	48.60 ± 10.14	47.10 ± 5.01	0.91	1.03	0.97	0.41
Maximum isokinetic concentric extension/flexion 180°/_S_	51.31 ± 7.21	51.65 ± 5.06	0.93	1.08	1.00	0.81
Maximum isokinetic concentric extension/flexion 300°/_S_	58.07 ± 10.48	55.64 ± 7.56	0.92	1.02	0.97	0.24
Maximum isokinetic eccentric extension/flexion 60°/_S_	162.70 ± 30.29	163.28 ± 22.50	0.98	1.01	1.00	0.92
Maximum isokinetic eccentric extension/flexion 180°/_S_	161.99 ± 26.06	163.73 ± 28.86	0.98	1.02	1.00	0.55

OR, odds ratios

The results of the logistic regression test showed that no significant relationship was found between isokinetic strength of knee extensor muscles at different rates of injury in young wrestlers (Table 3). However, wrestlers who had higher average strength to perform concentric extension movements at different speeds of 60, 180, and 300°/s and eccentric extension movements at 60°/s had significantly fewer injuries than other wrestlers (Table 3).

**Table 3:** Comparison logistic regression analysis between injured and non-injured wrestler (shoulder)

Variabbles	Non-injured	Injured	Lower	Upper	OR	*P* value
Maximum isokinetic concentric flexion 60°/_S_	1.43 ± 0.33	1.31 ± 0.34	0.04	222.70	3.24	0.58
Maximum isokinetic concentric flexion 180°/_S_	1.30 ± 0.32	1.14 ± 0.31	0.01	5.78	0.26	0.39
Maximum isokinetic concentric flexion 300°/_S_	1.35 ± 0.45	1.13 ± 0.37	0.02	14.26	0.62	0.76
Maximum isokinetic concentric extension 60°/_S_	1.79 ± 0.43	1.64 ± 0.43	0.01	14.84	0.41	0.63
Maximum isokinetic concentric extension 180°/_S_	1.58 ± 0.36	1.47 ± 0.38	0.04	211.77	3.17	0.59
Maximum isokinetic concentric extension 300°/_S_	1.49 ± 0.42	1.33 ± 0.38	0.04	48.60	1.45	0.83
Maximum isokinetic concentric extension/flexion 60°/_S_	80.98 ± 10.58	80.68 ± 8.94	0.95	1.04	0.99	0.89
Maximum isokinetic concentric extension/flexion 180°/_S_	83.54 ± 18.69	78.78 ± 13.38	0.95	1.01	0.98	0.21
Maximum isokinetic concentric extension/flexion 300°/_S_	90.51 ± 18.65	85.18 ± 12.39		1.00	0.97	0.14

OR, odds ratios

## Discussion

The study results showed no significant relationship between the isokinetic strength of flexor and extensor muscles of the knee at different angular speeds of 60, 180, and 300°/s, isokinetic strength of flexor and extensor muscles of the shoulder at different angular speeds of 60, 180 and 300°/s, the ratio of the strength of flexor to extensor muscles of the knee to the strength of flexor to extensor muscles of the shoulder in young elite wrestlers and the rate of injuries. We used speeds that were similar to the wrestling's movement patterns. In the wrestling, movements are performed at low, medium and high speeds. Therefore, these velocities were used to measure isokinetic strength. Based on the principle of chain reactions of the body, the optimal strength of the knee joint can protect the whole body against injury. It has been published recently that dysfunction in the ankle dorsiflexion can be recognized a risk factor for shoulder and elbow injuries in baseball athletes [26]. So, recording injuries to all parts of the body were conducted accordingly to look for possible links.

There are not many studies on the relationship between the isokinetic strength of knee flexor muscles and the rate of injuries in wrestlers and individual sports in general. Most similar studies have been conducted in team and ball sports. For example, Van Dyk et al. [27] showed that the isokinetic strength of quadriceps muscles at the speed of 60°/s could not predict hamstring injuries. Henderson et al. [28] reported that the isokinetic strength of extensor muscles of the knee of English Premier League football players at the speed of 180°/s could not predict hamstring injuries. Bakken et al. [19] also stated that the maximum quadriceps muscle torque of the players of 14 Qatari Premier League teams at the speed of 60°/s had no significant relationship with the rate of their injuries. Hietamo et al. [29] also stated that the isokinetic strength of quadriceps muscles in male and female basketball and football players cannot predict the rate of knee injuries.

Kim et al. [30] examined the effect of knee pain perception on muscle strength and reported that no significant differences in knee muscle strength of both the flexor and extensor muscles and muscle strength (hamstring/quadriceps ratio180°/s) were noted between the high and low groups. The pain which is a consequence of knee injuries could not significantly affect muscle strength in this study., while in our study, wrestlers who had higher average strength to perform concentric flexion at different speeds of 60, 180, and 300°/s, and eccentric flexion at different speeds of 60 and 180°/s suffered significantly fewer injuries than other wrestlers. The difference between the two studies could be due to that level of strength is a determining factor for sports injuries which can be independent of pain following an injury.

The non-functional evaluations performed by the isokinetic device can be considered as one of the reasons for the inability of these tests to predict injuries. The evaluation performed by this device is without weight-bearing, while all movements in wrestling are done with weight-bearing. The movements in wrestling are performed as a closed movement chain, while the test of this device is performed as an open movement chain [31].

According to the study results, none of the ratios of isokinetic strength of quadriceps hamstrings at 60, 180, and 300°/s could predict wrestlers' injuries. Bakken et al. [19] also stated that the ratio of isokinetic strength of H/Q muscles at different speeds of 60 and 300°/s could not predict the injuries of football players. Hietamo et al. [29] also reported that the ratio of isokinetic strength of H/Q muscles was not significantly related to knee injuries. The ratio of the strength of H/Q muscles has been used for more than 60 years to identify muscle imbalances, to investigate the stability of the knee joint, and to describe strength characteristics of the muscles around the knee. However, there is disagreement in using this index as a predictor of injury [32].

Ruas et al. (2019) stated that investigation of the ratio of the maximum torque of H/Q muscles cannot fully investigate neuromuscular factors affecting the rate of injuries. These researchers suggest that the ratio of antagonist to agonist muscles at different angles and angular speeds should be considered to effectively predict injuries. These researchers also suggested that factors such as torque development rate, muscle size, and muscle activation could be used to increase the effectiveness of injury prediction [32].

As in the present study, Andrew et al. [33] conducted a study on the shoulder of 22 rugby players and reported that the isokinetic strength of the muscles around the shoulder is not able to predict shoulder joint injuries. Forthomme et al. (2018) in a study measured the isokinetic strength of shoulder rotator cuff muscles of 108 handball players at different angular speeds of 60 and 240°/s concentric and 60°/s eccentric. The researchers reported that the maximum isokinetic torque was not able to predict the shoulder joint injuries at any of the speeds and contractions and that isokinetic strength ratios of flexor to extensor muscles and internal to external rotators were not able to predict injuries [34]. Vogelpohl et al. [35] measured the isokinetic strength of the shoulder muscles of 15 baseball players. They reported that isokinetic strength at different angular speeds of 60 and 180°/s could not be a factor in predicting their injuries. The injuries in wrestling occur at explosive movements and very high speeds, but the isokinetic device cannot simulate the speed of movements well [31].

### Limitations

Although this study is one of the first studies investigating the internal risk factors of individual sports such as wrestling, there have been limitations to the method of measurement and the selected tests. Most of the tests in this study were performed in a sitting and no weight-bearing position and the open chain, while the performance of wrestlers during competition and training is different. These tests were not practical in terms of routine actions or movements in wrestling. It seems that measurements can be more effective when bearing weight and in situations close to wrestling.

The role of the internal and external rotator cuff muscles of the shoulder joint in performing wrestling techniques is higher than that of flexor and extensor muscles, and perhaps it would be better to measure the strength of these muscles as well. In addition, the subjects of this study were from two Freestyle and Greco-​Roman styles. There is a different nature to the techniques of the two styles, however, we did not differentiate between Freestyle and Greco-Roman style wrestlers due to the reduction in sample size. We measured the dominant knee and shoulder only and consequently, we did not calculate ratios between the dominant and the non-dominant knee and shoulder.

## Conclusion

The isokinetic strength of the knee in flexion and extension movements cannot be effective in predicting wrestlers' injuries. Therefore, it is recommended to use this factor to identify at-risk wrestlers. Given that isokinetic strength factors of knee and shoulder muscles were not effective in predicting injuries, so these factors are not recommended for evaluating high-risk wrestlers. The study results also showed that none of the maximum torques measured in different muscle groups could predict wrestlers' injuries.

## Data Availability

The datasets generated during and analyzed during the current study are not publicly available due to ethical restrictions, however are available from the corresponding author on reasonable request.

## Abbreviation

H/Q: Hamstring to quadriceps
